# Effect of a price display intervention on laboratory test ordering behavior of general practitioners

**DOI:** 10.1186/s12875-021-01591-w

**Published:** 2021-12-03

**Authors:** Dennis M. J. Muris, Max Molenaers, Trang Nguyen, Paul W. M. P. Bergmans, Bernadette A. C. van Acker, Mariëlle M. E. Krekels, Jochen W. L. Cals

**Affiliations:** 1 MCC Omnes Centre for Diagnostics and Innovation, Sittard, The Netherlands; 2 Department of Family Medicine, CAPHRI Care and Public Health Research Institute, Maastricht University, PO BOX 616, 6200 MD Maastricht, The Netherlands; 3 Department of Clinical Chemistry, Zuyderland Medical Centre, Sittard, The Netherlands; 4 Department of Internal Medicine, Zuyderland Medical Centre, Sittard, The Netherlands

**Keywords:** Diagnostic tests, Healthcare costs, Test ordering rate, Primary care

## Abstract

**Background:**

Redundant use of diagnostic tests in primary care has shown to be a contributor to rising Dutch healthcare costs. A price display in the test ordering system of the electronic health records (EHRs) could potentially be a low-cost and easy to implement intervention to a decrease in test ordering rate in the primary care setting by creating more cost-awareness among general practitioners (GPs). The aim of this study was to assess the effect of a price display for diagnostic laboratory tests in the EHR on laboratory test ordering behavior of GPs in the Westelijke Mijnstreek region in the Netherlands.

**Methods:**

A pre-post intervention study among 154 GPs working in 57 general practices was conducted from September 2019, until March 2020, in the Netherlands. The intervention consisted of displaying the costs of 22 laboratory tests at the time of ordering. The primary outcome was the mean test ordering rate per 1.000 patients per month, per general practice.

**Results:**

Test ordering rates were on average rising prior to the intervention. The total mean monthly test order volume showed a non-statistically significant interruption in this rising trend after the intervention, with the mean monthly test ordering rate levelling out from 322.4 to 322.2 (*P* = 0.86). A subgroup analysis for solely individually priced tests showed a statistically significant decrease in mean monthly test ordering rate after implementation of the price display for the sum of all tests from 67.2 to 63.3 (*P* = 0.01), as well as for some of these tests individually (i.e. thrombocytes, ALAT, TSH, folic acid). Leucocytes, ESR, vitamin B12, anti-CCP and NT-proBNP also showed a decrease, albeit not statistically significant (*P* > 0.05).

**Conclusions:**

Our study suggests that a price display intervention is a simple tool that can alter physicians order behavior and constrain the expanding use of laboratory tests. Future research might consider alternative study designs and a longer follow-up period. Furthermore, in future studies, the combination with a multitude of interventions, like educational programs and feedback strategies, should be studied, while potentially adverse events caused by reduced testing should also be taken into consideration.

**Supplementary Information:**

The online version contains supplementary material available at 10.1186/s12875-021-01591-w.

## Background

Healthcare expenditures in the Netherlands have shown an expansive growth in recent years. In 2018, the Dutch healthcare costs exceeded the threshold of 100 billion euros for the first time. With a 7.1% increase in expenses in 2019, primary care is the second biggest riser in growing healthcare costs and is therefore proving to be a considerable contributor to the growth of total Dutch national healthcare expenditures [1, 2]. A vast source of rising primary healthcare costs is the expanding use of diagnostic testing [3–5]. Despite being an indispensable part of medical practice, about 20–30% of diagnostic tests have shown to be of limited clinical value, therefore contributing to the redundant growth of Dutch healthcare expenditures [3–8]. Consequently, diagnostic testing has become an attractive target for intervention to reduce future healthcare costs in the Netherlands, with the aim of preserving a long-term accessible and affordable Dutch health care system [3–9].

Over the past two decades, a variety of interventions has been implemented in efforts to influence general practitioners’ (GPs) diagnostic test ordering behavior. These interventions consisted of audits and feedback programs, educational sessions, guideline development, peer management and multidimensional techniques [4, 5, 7, 10–12]. Although the motivational, educational, and contextual factors that these interventions are based on appear to be significant factors in influencing ordering behavior, recent studies have shown inconsistent sustainability of effects. Furthermore, GPs have labeled these interventions to be labor intensive and time consuming, therefore motivating the need for alternative approaches [5, 7, 10, 12, 13]. One such low-cost and easy-to-implement alternative is the implementation of laboratory cost display in electronic health records (EHRs).

Displaying the costs of laboratory tests in the EHR entry system at the time of test ordering, is an easy to implement technique that would not interfere with current ordering processes. Moreover, cost display educates GPs by increasing their cost-awareness, and could potentially lead to a reduction in test ordering [3–7, 9, 14, 15]. While some studies have shown encouraging results, insufficient or methodological questionable data makes it difficult to draw strong conclusions [9, 14, 15], while in order to reduce healthcare costs without threatening quality of care, it is especially important to test these interventions in the primary care setting [5, 6, 9, 13, 15].

Therefore, with the aim of reducing the laboratory test ordering rate, we implemented the display of laboratory costs in the EHRs in a pre-post intervention study amongst GPs in one large geographically demarcated region in the Netherlands (Westelijke Mijnstreek). We hypothesized that displaying these costs at the time of ordering would create more cost-awareness amongst GPs, therefore causing a more cost-conscious test ordering behavior, consequently leading to a decrease in test ordering rate while eventually restricting the marked rise of total health care costs.

## Methods

### Study design and population

This single-arm observational study was conducted over a 6-month period from September 1st, 2019, until March 1st, 2020, in the Westelijke Mijnstreek, a region in the province of Limburg in the Netherlands. The total study population was composed of 190.427 enlisted patients from 154 GPs and 57 general practices in 2019 [16]. There were no specific in- or exclusion criteria for patients.

### Study setting

Data were retrieved from Medical Coordinating Center (MCC) Omnes. MCC Omnes is the purchasing organization for laboratory testing diagnostics in the Westelijke Mijnstreek region. From 2012, health insurers in the Netherlands introduced a budget ceiling for laboratory testing diagnostics. Due to this budget ceiling for laboratory testing diagnostics, MCC Omnes was forced to reduce costs or laboratory test ordering from GPs. As a result, MCC Omnes developed – with mutual agreement of medical specialists and GP’s – various instruments to achieve this reduction and has repeatedly experimented with several educational and feedback intervention strategies like project groups and extra in-service trainings, experimented with several educational and feedback intervention strategies like project groups and extra in-service trainings. Moreover, it provides a secured web application for electronic laboratory test ordering (Cyberlab), which approximately 95% of GPs in the region use [17]. GP-ordered laboratory testing in The Netherlands is covered by health insurances although every Dutch citizen has a deductible for the first 385 euro of health care costs in a year, with basic coverage such as GP consultations excepted.

### Intervention description

**Fig. 1:**
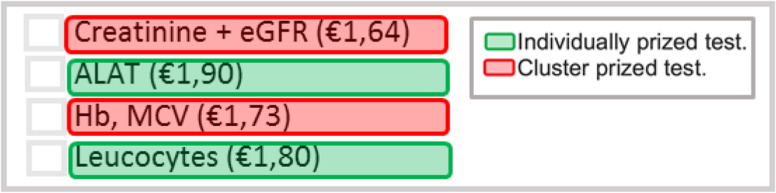
Display price information at the time of ordering in the Cyberlab laboratory test ordering screen

The intervention, which was implemented simultaneously for all participating GPs on September 1st, 2019, consisted of displaying the costs of 22 laboratory tests in the Cyberlab test ordering screen. These tests were selected by two independent GPs based on the available data on either high volume or high unit costs. Costs for nine of these tests were displayed individually per test; thrombocytes, leucocytes, ALAT, TSH, folic acid, vitamin B12, anti-CCP and NT-proBNP (see Fig. 1 for an example, marked in green). For the 13 remaining tests, costs were displayed in a variety of panels of tests. These panel priced tests consisted of glucose, creatinine, eGFR, Hb, MCV, sodium, potassium, free T4, total cholesterol, cholesterol-HDL ratio, LDL, HDL and triglycerides (see Fig. 1 for an example, marked in red). A complete overview of the costs for all individually or panel priced tests is included in the appendix (appendix 1). The displayed costs were based on the average set charge for laboratory tests (MCC Omnes 2019). All participants were informed by the introduction of price displays through email prior to the implementation of the intervention.

### Data collection and outcome assessment

To assess the changes in test ordering rates amongst GPs before and after the implementation of the price display, a pre-post intervention approach was used. Data from September 1st, 2017, until March 1st, 2019, were classified as the pre-intervention period. Moreover, this pre-intervention period was subdivided into Period 1, consisting of data from September 1st, 2017, until March 1st, 2018, and Period 2, consisting of data from September 1st, 2018, until March 1st, 2019. Furthermore, data from September 1st, 2019, until March 1st, 2020, were classified as the post-intervention period, henceforth also marked as Period 3. For the 22 specified laboratory tests, we determined the number of ordered diagnostic tests per month for the two predefined pre- and one post-intervention periods.

### Statistical analysis

Test ordering rates were calculated for the pre- and post-intervention periods, in terms of mean number of tests per 1.000 patients per general practice per month. These data were tested for normality by performing the Shapiro-Wilk test. We used a paired two sample t-test to calculate change scores between periods if data were distributed normally, and., a Wilcoxon signed rank-test if data were not normally distributed. Additionally, two subgroup analyses were made. First, similar analyses (i.e. differences in ordering rates for the pre- and post-intervention periods) were made for solely the nine individually prized tests. Secondly, per-test analyses were made for each of the individually prized tests. A *P*-value of < 0.05 was considered statistically significant. Statistical analysis was performed using IBM SPSS Statistics for Mac, version 26.0 (IBM Corp., Armonk, N.Y., USA).

## Results

### Total mean monthly test order volume

**Fig. 2:**
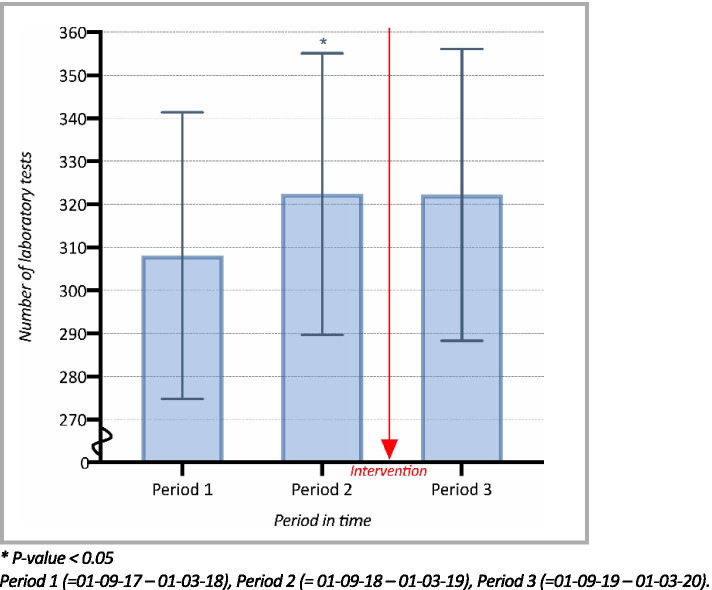
Mean monthly number of laboratory tests ordered per general practice, per 1.000 patients (95% CI)

Changes in mean monthly test order rates are presented in Fig. 2. During the pre-intervention period, the mean monthly test order rates showed a rising trend, with an 4.5% increase from 308.1 (95% CI; 274.8–341.4) in Period 1 to 322.4 (95% CI; 289.7–355.1) in Period 2. This change was statistically significant (*P* = 0.001). In Period 3, after the implementation of the intervention, an interruption in this rising trend was shown. The mean monthly test order rate decreased by 0.06% to 322.2 (95% CI; 288.3–356.1), therefore remaining more or less identical to the pre-intervention Period 2. However, this result was not statistically significant (*P* = 0.86).

### Effects of intervention for individually prized tests

**Fig. 3:**
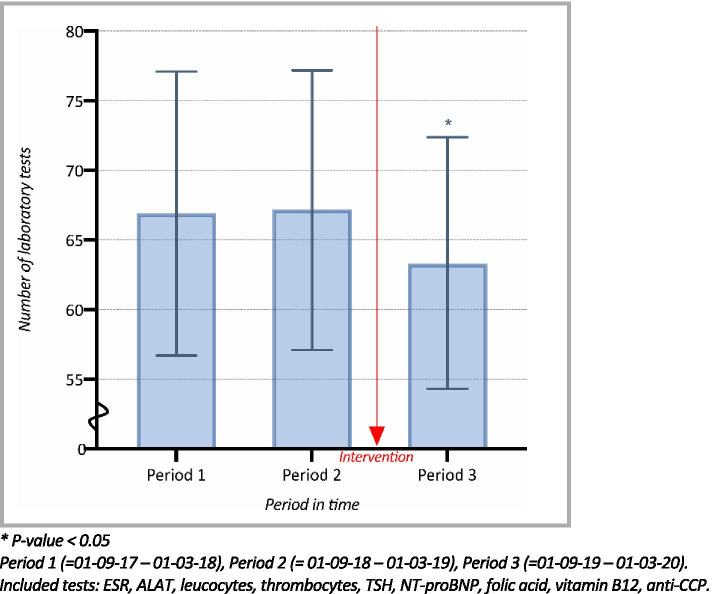
Mean monthly number of individually prized laboratory tests ordered, per general practice, per 1.000 patients (95% CI)

Additionally, a similar subgroup analysis was made which consisted solely of the nine individually prized laboratory tests. Changes in mean monthly test order rates are presented in Fig. 3. During the pre-intervention period, the mean monthly test ordered rates remained fairly constant with just a 0.4% increase from 66.9 (95% CI; 56.7–77.1) in Period 1 to 67.2 (95% CI; 57.1–77.2) in Period 2(*p* = 0.92). In Period 3, after the implementation of the intervention, the mean monthly test order rate declined to 63.3 (95% CI; 54.3–72.4), therefore showing a statistically significant (*P* = 0.01) decrease of 6.1% compared to pre-intervention Period 2.

**Fig. 4:**
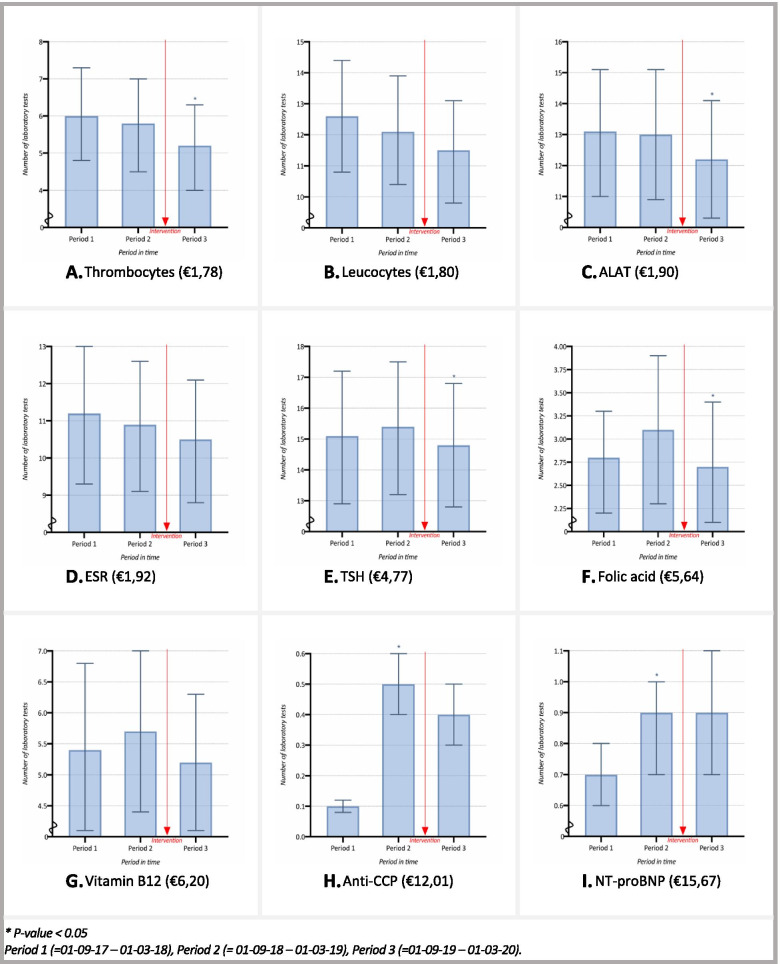
Per-test mean monthly number of laboratory tests ordered, per general practice, per 1.000 patients (95% CI), ranked by ascending costs

Changes in mean monthly test order rates per test are presented in Fig. 4A. For a certain group of tests (thrombocytes, leucocytes, ALAT and ESR), a decreasing trend was shown throughout time, as seen in Fig. 4A-D. These tests showed a decline from Period 1 to Period 2, varying from 0.5 to 4.5%, although none of these changes were statistically significant (*p* > 0.05). From Period 2 to Period 3 however, after the implementation of the intervention, the decline varied from 5.4 to 10.2%. Whereas these changes were not statistically significant for leucocytes and ESR (*P* > 0.05), as seen in Fig. 4B and D, thrombocytes and ALAT on the other hand showed a statistically significant change (*P* = 0.02 and *P* = 0.04, respectively), as seen in Fig. 4A and C.

Moreover, for a group of other tests (TSH, folic acid, vitamin B12, anti-CCP and NT-proBNP), a trend interruption was shown in Period 3, after the implementation of the intervention, as seen in Fig. 4E-I. These tests showed an increase from Period 1 tot Period 2, varying from 1.7% to a 126%. On the contrary, from Period 2 to Period 3, after the implementation of the intervention, an interruption in this rising trend was shown, with rates declining varying from 3.8 to 18%. These changes were statistically significant for TSH and folic acid (*P* = 0.04), albeit not for vitamin B12, anti-CCP and NT-proBNP (*P* > 0.05).

## Discussion

The implementation of a laboratory test price display at the time of ordering can constrain GP laboratory test ordering. With a rising trend in test ordering rate prior to the price display intervention, we found a noticeable interruption in this rising trend after the implementation of the price display, albeit not statistically significant. We found a statistically significant decrease in test ordering rate after implementation of the price display for the sum of all individually priced tests as well as for some of these tests individually (i.e. thrombocytes, ALAT, TSH, and folic acid).

These study findings suggest that the implementation of a price display at the time of ordering can in fact alter physicians behavior and constrain laboratory test ordering. This is in line with previous literature concerning this subject, in both primary care [3, 18] and secondary care [5, 9, 15] settings. However, there are also some studies, as well in both primary care [7, 14] and secondary care [6, 13, 14] settings, that show no effect of price display on laboratory test ordering. A number of possible explanations for this variability in results have been hypothesized. Firstly, one can only speculate about the influence of varying populations and differing diagnoses between primary and secondary care on test ordering behavior. For example, it is likely that in secondary care setting, patients are often sicker than in primary care setting, consequently making test ordering less discretionary, therefore showing less impact of a price display intervention [13]. Secondly, 90% of resident physicians in secondary care reported that redundant laboratory testing was often due to the practice habit of already ordering repeated daily laboratory test orders ahead on the patient’s day of admission. Therefore, physicians would not be presented with price display during decisions later on, when the necessity of further test ordering might be most questionable and therefore the effect of price display could be of most value [6]. Lastly, the aforementioned studies that showed no impact of price display, all consisted of a singular intervention. So far, few single interventions have clearly resulted in a sustained decrease in laboratory ordering rate. Therefore, a variety of combinations of interventions has been explored in recent years in efforts to influence practitioners diagnostic test ordering behavior more thoroughly. Besides price display, these interventions consisted of audits, educational sessions and feedback programs [8, 10–12, 19, 20]. In conclusion, these studies suggested that combining a multitude of interventions and reinforcing them over time, is favored in order to perpetuate a sustainable reducing effect on future laboratory test ordering [7, 8, 10, 11, 13, 20].

Interestingly, MCC Omnes developed – with mutual agreement of medical specialists and GP’s – various instruments to achieve this reduction and has repeatedly experimented with several educational and feedback intervention strategies like project groups and extra in-service trainings, and with several educational and feedback intervention strategies like project groups and extra in-service trainings. A successfully proven intervention has been diagnostic testing peer audit and feedback groups of local quality improvement collaboratives [11, 21, 22], which were implemented, especially in period 1. During these meeting of local quality improvement collaboratives, a group of local GPs receive education and feedback about their ordering behavior, with the purpose of achieving a more favorable, cost-effective test ordering behavior [16]. And while other interventions, such as these meetings and other unmeasured influences over time could have a confounding effect, our study results show that while diagnostic testing peer audit and feedback groups have been implemented in Period 1, there was still an increasing trend in ordering rate to be seen towards Period 2. On the contrary, after the addition of the price display, in Period 3, a noticeable interruption (Fig. 2) and even a clear decrease (Fig. 3) was shown in this previously rising trend. This strongly suggests that a price display intervention could be a low-cost and simple yet valuable addition to combine with educational and feedback intervention strategies like the GP Quality Improvement Collaborative meetings. Therefore, in order to reduce the expanding use of laboratory testing in the primary care setting, future studies should focus on the effectiveness of combining a price display intervention with educational programs and feedback strategies. Ongoing local quality improvement collaboratives could have had a confounding effect on the results, yet Lastly, previous literature has shown no differences in the rates of hospital admissions or visits to the emergency room following a price display intervention [5, 12, 15]. While our research did not study these aspects, it is important to note that future research should also take the possibility of these potentially adverse events, caused by reduced testing, into account.

There are several limitations to our study. Firstly, our study has a relatively short follow-up period of 6 months. This makes it difficult to draw strong conclusions about the long-term effects of the intervention. Initially, it was planned that our study would provide a more extended follow-up period of 12 months, which would have been preferable to gain a more accurate insight into the sustainability of effects. However, the unexpected interference of the COVID-19 pandemic in the Netherlands in March 2020 caused a complete disruption of routine Dutch healthcare, therefore leading to unreliable study data from March 2020 onwards. Consequently, we were forced to restrain the follow-up period of our study to just 6 months. Secondly, we chose to use a pre-post intervention design and potentially this design could threaten the causal interpretation of observed effects [23, 24]. Inevitably, a randomized controlled trial (RCT) design was not achievable in our study, since every intervention implemented into diagnostic test ordering system Cyberlab would automatically be implemented for all GPs in the whole region, therefore leaving no room for both an intervention and a control group. Thirdly, as mentioned in the introduction, for some tests the costs were displayed individually per test, while for other tests costs were displayed in panels of tests (Fig. 1). For our study period, we were only able to obtain data per test, and not per panel, and as a consequence, calculating the ordering rate per panels of tests was not possible. Hence, in order to draw even more accurate conclusions, future studies should definitely consist of not only data per test but also of data per cluster. While our hypothesis was that the largest effects would be expected for the mostly costly tests, this was observed for TSH and folic acid, yet not for vitamin B12, anti-CCP and NT-proBNP. One explanation could be that some test ordering is more patient driven, such as vitamin B12, and that an intervention targeted at physicians will have limited impact. Lastly, it is important to distinguish between redundant and necessary testing We found a statistically significant decrease in test ordering rate after implementation of the price display. However, there is a possibility that quality of care could be compromised because of reducing necessary testing due to this intervention. Future studies must take this in consideration.

There are also several strengths to our study. Firstly, one of the major strengths that our study offers is that it uses a large study population, consisting of 154 GPs and 57 general practices. There is no reason to believe that this large study population differs from the rest of the Dutch primary care physician population, therefore our study provides a high external validity. Secondly, whereas previous international studies have shown positive results for price display interventions in the clinical as well as in the primary care setting, this is, to our knowledge, the first Dutch study that uses the EHR to implement a price display intervention at the time of ordering in the primary care setting. Thirdly, whilst displaying the costs of tests educates GPs by increasing their general cost-awareness, the fact that prices are shown at the time of ordering also empowers GPs in the act of informed decision making during patient encounters [3]. Lastly, the price display that we implemented in the EHRs is an easy to implement technique that requires minimal resource outlay and does not intervene with current ordering processes, therefore making it an attractive tool for GPs [3, 5, 13]. Moreover, in the future, it could easily be adapted to the dynamic landscape of annually changing healthcare costs, therefore making it not only a simple and effective, but also a sustainable tool to help reduce the expanding use and costs of diagnostic testing in the future.

The results of our study carry broad implications. To start with, our results are consistent with previous literature in confirming the increasing trend in test ordering rate in recent years. This annually expanding use of diagnostic tests has shown to be a vast contributor to the expansive growth of healthcare expenditures in the Netherlands [3–5]. Aside from this financial burden, the inappropriate and expanding use of laboratory tests also carries the risk of overuse, therefore providing a possibly higher level of false-positive results. This in return may result in the cascade of unnecessary further diagnostics or even overtreatment, eventually having a potentially harmful and threatening effect on the overall quality of care [8, 11, 19, 25]. The increasing trend in laboratory test ordering in our study, underlines that diagnostics remain an important target for intervention to reduce future healthcare costs in clinical setting as well as in primary care setting [3–15, 20].

## Conclusions

In conclusion, our study suggests that displaying the costs of laboratory tests in the EHRs to GPs at the time of ordering, is a simple tool that can alter physicians behavior and constrain laboratory test ordering. We observed a rising trend in overall test ordering rate prior to the intervention. Our study results show that for the total mean order volume, the price display intervention was associated with a noticeable, albeit not statistically significant, interruption in this rising trend. A further subgroup analysis even showed that the price display was associated with an obvious and statistically significant decrease in test ordering rate. Future studies on this subject might consider alternative study designs that consist of a longer follow-up period. Furthermore, future research should study the effectiveness of combining and reinforcing a price display intervention with educational programs and feedback strategies, while also focusing on the possibility of potentially adverse events caused by reduced testing.

## Supplementary Information

**Additional file 1 **: **Appendix 1**. Overview of costs of per individual test or panels of test.

## Data Availability

The datasets used and/or analysed during the current study are available from the corresponding author on reasonable request.
