# Supplementary material for: Effect of a price display intervention on laboratory test ordering behavior of general practitioners
Source: BMC Fam Pract. 2021 Dec 3;22:242. doi: 10.1186/s12875-021-01591-w (PMC8639847; doi:10.1186/s12875-021-01591-w)
Supplement: Supplementary file 1 — Additional file 1 : Appendix 1. Overview of costs of per individual test or panels of test. [file 12875_2021_1591_MOESM1_ESM.docx]

Appendix

Appendix 1. Overview of costs of per individual test or panels of test

| **Individually priced tests** | **Costs  (€)** |  | **Panel of priced tests** | **Costs (€)** |  | **Individually + panels of**  **priced tests** | **Costs (€)** |
| --- | --- | --- | --- | --- | --- | --- | --- |
| Thrombocytes | 1,78 |  | Creatinine + EGFR | 1,64 |  | Potassium (K) | 1,63 |
| Leukocytes | 1,80 |  | Hb, MCV | 1,73 |  | Glucose | 1,64 |
| ALAT | 1,90 |  | Sodium(Na), Potassium(K) | 3,28 |  | Sodium (Na) | 1,65 |
| ESR | 1,92 |  | TSH (+ Free T4) | 4,77 |  |  | |
| TSH | 4,77 |  | **H103**: Glucose, Creatinine + eGFR, LDL | 7,30 |  |  |  |
| Folic acid | 5,64 |  | **H065**: Hb, K, TSH (+ Free T4) | 8,13 |  |  |  |
| Vitamin B12 | 6,20 |  | **H104**: Glucose, Kreatinine +eGFR, Cholesterol, HDL+ratio, LDL, Triglycerides | 9,71 |  |  |  |
| Anti-CCP | 12,01 |  | **H037**: Hb, MCV,  + follow-up | 45,49 |  |  |  |
| NT pro-BNP | 15,67 |  |  | |  |  |  |
